# IgM and IgG Epitope Mapping of the Porin Outer Membrane Protein-2a from *Brucella abortus*: Potential Biomarkers for Detecting Exposure to Brucellosis

**DOI:** 10.3390/ijms27125341

**Published:** 2026-06-13

**Authors:** Armando F. Noguera, Guilherme C. Lechuga, Paloma Napoleão-Pêgo, Joao P. R. S. Carvalho, Larissa R. Gomes, Andreia Carneiro da Silva, Marianne Melo Monnerat, Flavio R. da Silva, Salvatore G. De-Simone

**Affiliations:** 1 Center for Technological Development in Health (CDTS)/National Institute of Science and Technology for Innovation in Neglected Population Diseases (INCT-IDPN), Oswaldo Cruz Foundation (FIOCRUZ), Rio de Janeiro 21040-900, Brazil; nogueraunig@fiocruz.br (A.F.N.); guilherme.curty@fiocruz.br (G.C.L.); paloma.pego@fiocruz.br (P.N.-P.); larissagomesr@gmail.com (L.R.G.); flaviorochafiocruz@gmail.com (F.R.d.S.); 2 Technological Center of the Marine Corps (CTecFN), Brazilian Navy, Rio de Janeiro 21010-076, Brazil; andreia.carneiro@marinha.br (A.C.d.S.); marianne.manerat@marinha.br (M.M.M.); 3 Program of Post-Graduation on Science and Biotechnology, Department of Molecular and Cellular Biology, Biology Institute, Federal Fluminense University, Niterói 22040-036, Brazil; joao.rangel@fiocruz.br; 4 Program of Post-Graduation on Parasitic Biology, Oswaldo Cruz Institute, FIOCRUZ, Rio de Janeiro 21040-900, Brazil

**Keywords:** brucellosis, *Brucella abortus* infection, transmembrane protein, Omp-2a, IgM and IgG epitope mapping, ELISA-peptide, diagnostic

## Abstract

Brucellosis is a globally prevalent zoonotic disease affecting both humans and animals. Its nonspecific clinical manifestations often complicate diagnosis, underscoring the need for reliable laboratory confirmation. Traditional serological assays, though widely used, suffer from limitations such as inconsistent sensitivity and false-positive results. To address these challenges, this study mapped IgM and IgG epitopes of the Brucella Omp-2a protein using sera from infected patients. Epitope identification was performed through SPOT synthesis on cellulose membranes, followed by assessment of potential cross-reactivity using peptide database analysis and ELISA validation. Three major IgM and seven IgG linear B-cell epitopes were identified, six of which demonstrated strong reactivity in peptide-ELISA. Importantly, no significant cross-reactivity with proteins from other human pathogens was detected. Two chimeric multi-epitope peptides, composed of 50 and 60 amino acids and integrating Brucella-specific IgM and IgG epitopes, exhibited excellent diagnostic performance in ELISA, achieving near 100% sensitivity and specificity. These findings support the potential of synthetic peptides as reliable and cost-effective alternatives to native antigens in serological assays. Further validation in larger, geographically diverse cohorts will be essential to confirm their diagnostic robustness and facilitate their integration into routine brucellosis diagnostics.

## 1. Introduction

Brucellosis is one of the most common endemic zoonotic diseases, affecting both animals and humans in many countries. Despite its impact, the disease remains neglected in human medicine, and no adequate nationwide control or surveillance programs have been implemented [1,2,3].

Of the twelve known Brucella species, only *B. melitensis*, *B. abortus*, *B. canis*, and *B. suis* (except biovar 2) are associated with human infection. These pathogens are facultative intracellular bacteria [4,5].

Brucellosis is considered an occupational disease. It primarily affects individuals who work with animals or animal products, particularly in agricultural regions where livestock farming and land cultivation are predominant.

The clinical symptoms in humans are nonspecific and may be confused with other febrile illnesses [6,7]. Common manifestations include undulant fever, back pain, headache, joint pain, fatigue, myalgia, weight loss, loss of appetite, depression, and night sweats. Severe cases may progress to arthritis, orchitis, endocarditis, hepatomegaly, or splenomegaly [8,9,10].

Transmission usually occurs from infected animals to humans through contaminated fluids and discharges such as blood, vaginal secretions, placental fluids, and aborted fetuses [11]. Human-to-human transmission is rare, but sexual contact and breastfeeding can facilitate the spread of the bacteria [12]. Infection may also occur through cuts, conjunctival inoculation, skin abrasions, or inhalation of infectious aerosols [13,14]. Major risk factors include occupational exposure, direct contact with sick animals, and the consumption of contaminated milk or dairy products [15,16].

The prevalence of brucellosis in livestock, particularly in low- and middle-income countries, causes major socioeconomic and public health challenges [17,18,19,20]. In some regions, incidence continues to rise due to a lack of awareness [21].

Early diagnosis in both humans and animals is essential for controlling the disease. Laboratory essays include culture, serology, and molecular techniques [22,23]. PCR-based tests and serological markers are sensitive and widely used; however, culture remains the “gold standard” for diagnosis due to its reliability in clinical and public health settings [24].

In endemic regions, serological tests are still the most common diagnostic tools because they are inexpensive, easy to use, and reliable for excluding infection. Nucleic acid amplification assays offer high sensitivity and specificity, providing rapid diagnosis [20,25,26]. However, patients who appear clinically cured may remain PCR-positive for extended periods [27]. Thus, culture and serological testing remain essential for diagnosis and monitoring until standardized commercial molecular tests become available.

Over the years, several attempts have been made to develop Brucella vaccines [28,29,30,31,32,33]. Despite these efforts, controlling the disease in both humans and animals remains challenging [27]. Rapid and specific diagnostic methods are therefore still crucial, especially in humans, since infected animals must be slaughtered to prevent spread [34,35].

Classical serological tests such as the tube agglutination test [36,37], Rose Bengal plate test [38,39], and complement fixation test [40], along with commercial ELISA kits [41], detect antibodies to cell wall polysaccharide antigens of smooth Brucella strains. These tests, however, may cross-react with related bacteria [42,43,44] and cannot distinguish infected from vaccinated animals.

In recent decades, research has focused on immunoreactive and pathogen-specific protein antigens. Studies have tested full recombinant proteins and chimeric cell wall proteins as candidate antigens for serological diagnosis in humans [45,46,47,48,49] and animals [37,50,51,52,53]. Yet, the reported specificity and sensitivity of protein-based assays remain inconsistent and sometimes contradictory [27,54].

This study aimed to identify linear B-cell epitopes of the *B. abortus* Omp-2a protein recognized by IgM and IgG. The goal was to develop multiepitope polypeptides for use in rapid IgM/IgG diagnostic assays.

Functionally, Omp-2a is part of the alphaproteobacterial porin family and contributes to osmotic homeostasis and to the bacterium’s adaptation to intracellular environments, including survival within macrophages. Variations in Omp-2a expression have been linked to immune evasion, complement resistance, and intracellular persistence. Moreover, Omp-2a influences host cytokine responses and helps shape the balance between Th1- and Th2-type immunity, an equilibrium that is critical for the chronicity of brucellosis.

Immunologically, Omp-2a is a strongly immunogenic antigen recognized early by the host immune system. Comparative analyses of Omp-2a and Omp-2b reveal subtle but immunologically relevant differences in epitope recognition and cross-reactivity. Although both share structural and functional features, Omp-2a tends to elicit stronger and more durable antibody responses, likely due to greater exposure epitopes. This characteristic makes Omp-2a an attractive target for the development of diagnostic assays capable of distinguishing natural infection from vaccination-induced responses.

Beyond its diagnostic relevance, Omp-2a has also been evaluated as a vaccine antigen. These findings reinforce the role of Omp-2a as a key antigenic determinant of *B. abortus* and a strategic candidate for the rational design of safer and more effective brucellosis diagnoses.

## 2. Results

### 2.1. Epitope Mapping of the Omp-2a

**Figure 1:**
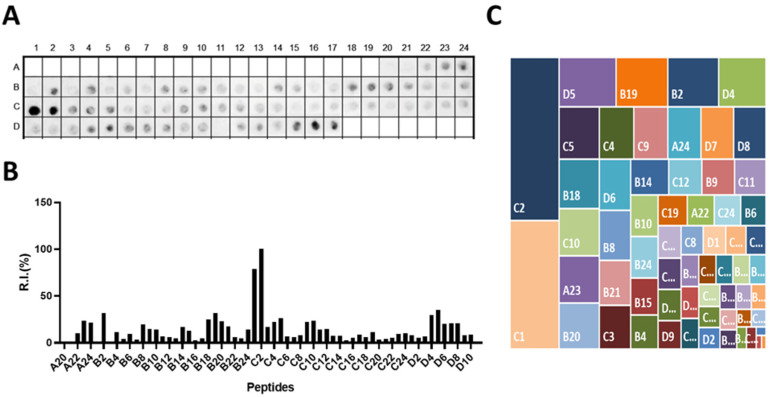
Mapping of linear IgM B-cell epitopes of the porin protein Omp-2a (Q44620; 321 aa) of *B. abortus* (biovar 1; strain 9-941). A library of 163 peptides, each 15 amino acid residue long with a 10-residue overlap, was tested for IgG reactivity against a pool of sera from patients diagnosed with Brucellosis (n = 15). (**A**) Immunoreactivity spot-membrane and (**B**) intensity of the chemiluminescent signal. (**C**) Analysis of hierarchical epitope recognition. The amino acid sequence of the peptides synthesized and their position on the membrane are shown in Table S2.

**Figure 2:**
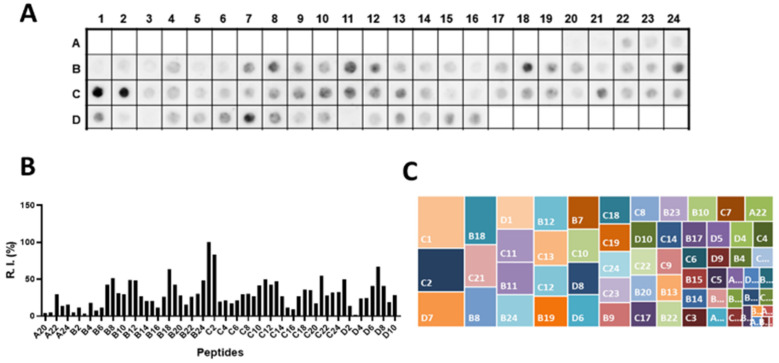
Mapping of linear IgG B-cell epitopes of the porin protein Omp-2a (Q44620; 321 aa) of *B. abortus* (biovar 1; strain 9-941). A library of 163 peptides, each 15 amino acid residues long with a 10-residue overlap, was tested for IgG reactivity against a pool of sera from patients diagnosed with Brucellosis (n = 15). (**A**) Immunoreactivity spot-membrane and (**B**) intensity of the chemiluminescent signal. (**C**) Analysis of hierarchical epitope recognition. The amino acid sequence of the peptides synthesized and their position on the membrane are shown in Table S2.

IgM and IgG linear B epitopes of the full Omp-2a (321 aa) protein recognized by the patient’s sera were identified using a library of 163 synthetic peptides covalently linked to a cellulose membrane (Figure 1A and Figure 2A) and developed as described before. Panel B shows the chemiluminescence intensity measurement using the patient’s serum, and Panel C shows the hierarchical distribution of the peptides according to their reaction intensity. For IgM, the most reactive peptide was C1, followed by C2, D5, and others. For IgG, the most reactive peptides were C1, C2, D7, and B18.

The intensities were normalized using the reactivity of the positive control as 100%. The ratio of the synthesized peptides and their respective positions in the membranes is shown in Table S2.

The overlapping sequences of the positive peptides defined 10 epitopes for Omp-2a, comprising 3 IgM and 7 IgG epitopes recognized by the patient’s sera pool (Table 1).

### 2.2. Shared Epitopes and Selection of Specific Brucella sp. Epitopes

**Figure 3:**
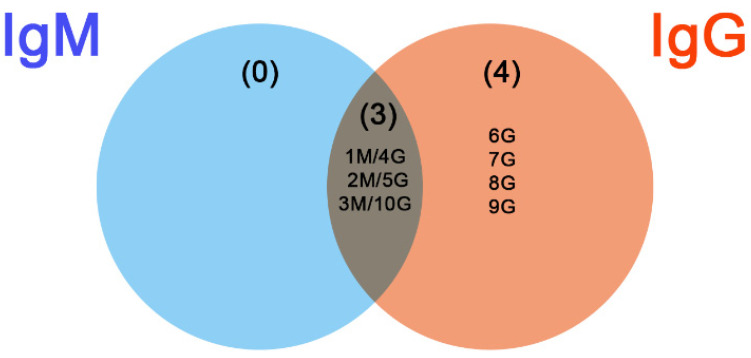
Venn diagram of the IgM and IgG common epitopes.

Three common structural overlapping epitopes recognized by both IgM and IgG were identified. These include the sequence NNSRHDGQYG, shared by epitopes Omp-2a/1M and Omp-2a/4G; NGFSAVIALE, found in Omp-2a/2M and Omp-2a/5G; and KFGGE, present in Omp-2a/3M and Omp-2a/10G (Figure 3). The remaining four IgG epitopes (6G, 7G, 8G, and 9G) were IgG isotype-specific and did not overlap with IgM epitopes.

Regarding specificity based on sequence database analysis, only the 3M and 10G epitope was exclusively found in the *Brucella* genus, indicating genus specificity, i.e., are present in Omp-2a of the structure of *Brucella* spp. but absent in other bacteria such as *E. coli*, *Shigella boydii*, and *Yersinia* spp. The other eight epitopes exhibited sequence homology with proteins from various non-pathogenic genera, including *Rhodotorula*, *Bartonella*, *Ochrobactrum*, and *Falsochrobactrum* (Table 1). However, none of these genera are known to include human pathogens.

**Figure 4:**
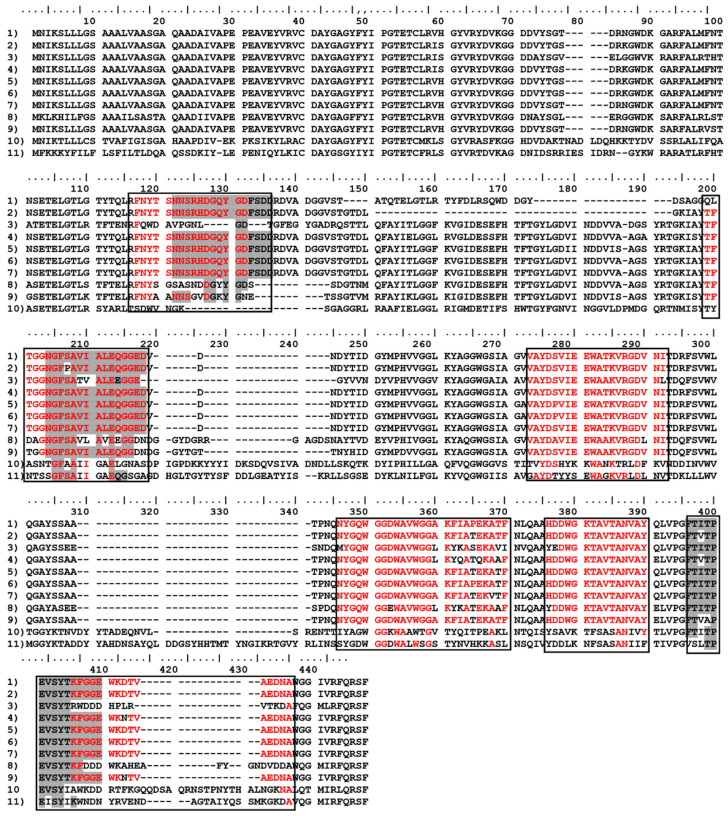
Sequence alignment of outer membrane proteins-2a. The alignment was carried out using ClustalW and included the following proteins: (1) Q44620 (*Brucella abortus*), (2) A5VPH9 (*B. ovis*), (3) Q45325 (*B. neotomae*), (4) A9MA14 (*B. canis*), (6) P0DI93 (*B. suis*), (6) Q7CNU3 (*B. melitensis)*, (7) A6X2A5 (*B. anthropi*), (8) (*Ochrobactrum anthropi)*, (9) A0A316JA34_9 (*Falsochrobactrum shanghaiense*), (10) Q45324 (*B. neotomae*), (11) YY3 (*Bartonella tamiae*). The regions marked in red rectangles indicate 10 epitopes present in *Brucella* sp. but absent in *E. coli* and *S. boydii*. The alignment follows the specified numbering order.

Figure 4 presents the alignment results of the *B. bovis* Omp-2a sequence with autotransporter proteins from various genera, demonstrating a general identity of 70% or more. In the figure, the colored rectangles demarcate only the unique linear B-epitopes (either IgM or IgG) that have been identified. No correlation was identified with *Yersinia* sp. proteins.

### 2.3. Structural Studies

**Figure 5:**
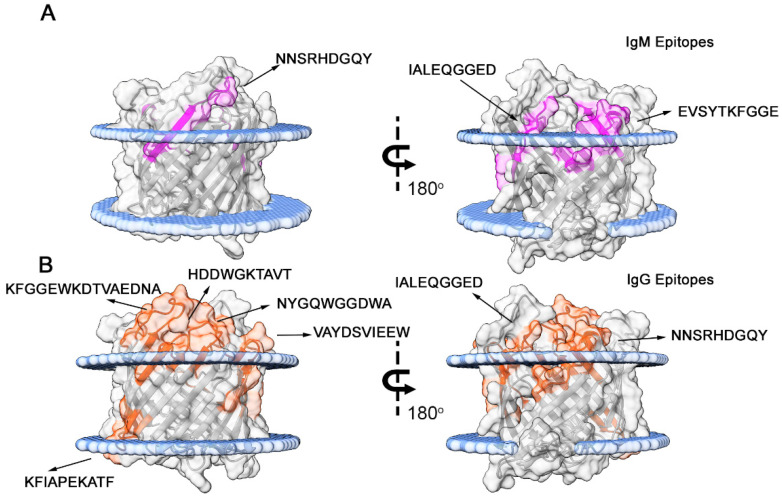
Three-dimensional structure predicted of Omp-2a from *B. abortus* showing the locations of immunodominant IgM (**A**) and IgG (**B**) epitopes. The structural model of Omp-2a (UniProt ID: Q44620) was generated using the I-TASSER server, with the toxin ABC protein complex from *Yersinia entomophaga* serving as the template.

The secondary structure elements of Omp-2a (Q44620) were identified using the TMpred tool, as described in the previous section. The protein has 11 alpha-helical transmembrane (TM) and two conserved domains (signal peptide and autotransporter) and tandem repeats, indicating that Omp-2a is an authentic OmpA (Figure 5) but shares no sequence homology with any other porin and a higher number of negatively changed residues in the exposed loops than Omp-2b [55].

The Omp-2a 3D model was constructed by homology modeling using as a template the available data toxin (ABC protein complex) *Yersinia entomophaga*, which presented the highest “score”. The models generated by the I-Tasser server presented reliability parameters with C-score values of 0.48 and RMSD = 8.6 (±4.5 Ǻ). The 3D model, illustrating the spatial distribution of epitopes, is shown in Figure 5. The amino acids that define the 10 (IgM and IgG) epitopes were located and marked on the model of secondary structure.

From this analysis, we can conclude that all epitopes are facing the external surface of the protein and are thus accessible to the immune system. All are “linear” structures with “coil” characteristics of linear B epitopes.

The protein presents 16-beta-barrel sheets, with large surface-exposed loops, that form a TM pore at the center of each barrel. The pore is partially occluded by a peptide loop that folds into the pore lumen. The larger pore formed by Omp-2a may be advantageous for intracellular growth when the bacterium competes with the host cell for nutrients whose concentration is particularly low within the phagosome.

### 2.4. Enzyme Immunoassay with Human Serum

**Figure 6:**
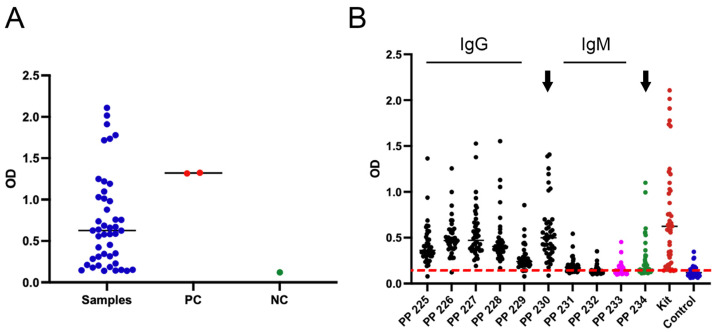
The cohort of sera from patients with Brucellosis was meticulously evaluated using a commercial ELISA (**A**) and detection of IgG antibodies. The specific responses were then validated against the synthetic peptides corresponding to the immunodominant IgG and IgM peptide/epitopes (**B**). Dashed line indicates the cut-off point (OD = 0.13 for B assays). Graph B shows the OD of every MAP4 single peptide (PP225-PP229, fifteen residues) reactive with IgG and IgM (PP231-PP233, fifteen residues) antibodies from the brucellosis infected patient’s sera and healthy individual as control (1:50 dilution). Peptides PP230 (IgG, sixty residues) and PP234 (IgM, fifty residues) represent chimeric peptides. Serum from healthy individuals in B was used as a control and no cross reactivity was detected (cut-off-point 0.13), a crucial element that ensures the validity and reliability of our findings. In Panel A, PC and PN indicate positive and negative controls, respectively. The positive controls are ready-to-use, including in the commercial kit. NC is a healthy serum used as an internal negative control.

From the sera of forty Brucellosis suspect patients, nineteen were identified as positive with a higher optical density (OD) using a commercial kit and were included in this study. The results are depicted in Figure 6A. Among the nineteen analyzed sera, only 1–3 did not exceed the cutoff values for all eight peptides analyzed (PP225-PP229 and PP231-PP233), whereas sera from twenty-one apparently healthy individuals consistently fell 3–5 units above the cutoff point (Figure 6B).

Cutoffs for each peptide (PP225-PP227 and PP231-233) were established using statistical analysis with ROC (Receiver Operating Characteristic) curve (Figure S1), as detailed in Table S3. The ROC curves illustrate the diagnostic test’s discriminatory ability across different cutoff values, highlighting the trade-offs between optical sensitivity and specificity. The points closest to the top left corner of the ROC curve indicate the most effective diagnostic thresholds.

Sensitivity, defined as the proportion of true positive results (TP) relative to all affected patients (TP + false negative [FN]), ranged from 0.88 to 0.95, indicating a strong statistical correlation between sera of patients with Brucellosis and healthy individual sera results.

Specificity, representing the proportion of true negatives (TN) among controls (TN + false positive results [FPR]) ranged from 90% to 95%. The control group analyzed in the ROC curve analysis consisted of healthy individuals, demonstrating that the in-house ELISA test peptide exhibits 90–95% specificity for the diagnosis of Brucellosis in humans, a similar value obtained by the commercial kit.

ROC curves provide quantitative assessments of diagnostic test accuracy, with the area under the curve indicating overall efficiency. Peptide PP225-PP228 and P230 demonstrated higher immunogenicity (90% specificity and 94% sensitivity) (Table S2).

## 3. Discussion

Brucellosis remains difficult to diagnose, particularly during the acute phase, due to the limited sensitivity, specificity, and interpretability of conventional serological assays [56]. Although ELISA-based detection of IgM and IgG antibodies is widely used, antibody persistence after infection often complicates clinical interpretation and hinders accurate disease staging [57,58]. To address these challenges, our study investigated epitope-level immune responses as a strategy to enhance diagnostic precision. By identifying epitopes associated with active or recent infection, we aimed to improve both sensitivity and stage-specific accuracy.

We focused on the Brucella Omp-2a protein, a membrane-associated antigen known for its strong immunogenicity [59]. Peptide array screening revealed ten linear B-cell epitopes—three IgM-reactive and seven IgG-reactive—that showed specific reactivity with sera from infected patients (Table 1). The limited number of IgM epitopes likely reflects isotype switching among individuals sampled during later stages of infection or following treatment. Notably, three IgM epitopes overlapped with IgG epitopes, suggesting that certain antigenic determinants elicit persistent humoral responses across disease stages. Conversely, IgG-exclusive epitopes may represent markers of late or memory-phase immunity, underscoring the diagnostic advantage of integrating early (IgM) and late (IgG) epitopes in serological assays.

Cross-reactivity analysis demonstrated that most epitopes were highly specific to Brucella, reinforcing their diagnostic value. However, partial sequence homology with Bartonella tamiae was observed, highlighting the need for selective epitope inclusion to minimize false-positive results. The structural organization of Omp-2a, a β-barrel with exposed surface loops, likely accounts for the accessibility and immunodominance of these epitopes [60,61,62], facilitating efficient antibody binding [63].

Understanding the kinetics of humoral responses is crucial for refining diagnostic markers. In human brucellosis, IgM typically appears within the first week of infection, peaks around the second month, and gradually declines, whereas IgG emerges after approximately two weeks and peaks between six and eight weeks. Although IgG responses more accurately reflect disease progression, the isolated presence of either IgM or IgG can complicate interpretation [64,65]. Therefore, markers capable of distinguishing early from late infection phases are essential for accurate diagnosis.

In our cohort, the scarcity of IgM epitopes likely reflected ongoing isotype switching, a hallmark of adaptive immunity that favors high-affinity IgG production over transient IgM. Interestingly, three IgM epitopes (Omp-2a/1M, 2M, and 3M) overlapped with IgG epitopes (4G, 5G, and 10G), indicating long-term recognition of these antigenic regions. In contrast, four IgG-specific epitopes (6G–9G) may correspond to late-stage or memory-associated immune responses. This distribution supports their differential application as markers of acute versus chronic or convalescent infection.

Validation of the immunodominant epitopes by ELISA confirmed their strong reactivity with patient sera. Four epitopes with no detectable homology to unrelated microorganisms demonstrated excellent diagnostic performance. Among these, sequences such as PP225–PP229, located on surface-exposed loops, were recognized by both IgM and IgG, suggesting durable and broadly reactive immune recognition represent and promising candidates for highly specific serological assays and for the design of synthetic immunogens. Conversely, epitopes 2M/5G and 7G exhibited cross-reactivity with *B. tamiae* proteins, rendering them unsuitable for universal diagnostic use.

Although Omp-2a and its homolog Omp-2b share similar structural frameworks, they display distinct epitope repertoires, as demonstrated in this study. Among the identified determinants, three epitopes (1M/4G, 2M/5G, and 3M/10G) were recognized by both antibody isotypes, while five were exclusively IgG-reactive. This pattern highlights their potential utility for improving IgM/IgG- or IgG-only assays, facilitating stage discrimination and enhancing diagnostic accuracy.

The approach used in our study delineates distinct IgM and IgG determinants of the Omp-2a that can refine serological detection of brucellosis but also provides a conceptual framework for vaccine development and a deeper understanding of *Brucella*-induced humoral immunity [66,67].

## 4. Materials and Methods

### 4.1. Human Sera

In this study, blood samples from 40 patients diagnosed with Brucellosis, confirmed by a commercial ELISA (Serion Brazil, Curitiba, Pr, Brazil), were analyzed. Patients were selected based on definitive positive ELISA test results, and the dataset was refined by excluding individuals with co-infections or immunosuppressive conditions to minimize bias. The cohort included patients at various clinical stages of Brucellosis, ensuring a broad representation of disease progression. Sera were obtained between 2012 and 2022 by the Central Public Health Laboratory (LACEN) of Rondônia and the Infectiology Service at the Oswaldo Cruz Policlinic in Rondônia, Brazil. The other twenty-one serum samples used were from healthy individuals obtained from the blood bank donor HEMORIO (Arthur de Siqueira Cavalcanti State Institute of Hematology, Rio de Janeiro, Brazil). For this study, patients’ identities were kept anonymous. The study was conducted in accordance with the Declaration of Helsinki and approved by the Ethics Research Committee of Faculty of Medicine/Federal Fluminense University (protocol code CAAE: 46304621.8.0000.5243 on 23 April 2021).

### 4.2. Spot Synthesis

Two libraries of 163 peptides, each covering the entire sequence of Omp-2a (UniProt Q44620) from *B. abortus* (biovar 1, strain 9-941), were synthesized. These peptides were 15 residues in length, with a 10-residue overlap, and were obtained using a synthesizer (Auto-Spot ASP222, Intavis Bioanalytical Instruments AG, Köln, Germany) and the F-moc (9-fluorenylmethoxy carbonyl) strategy, as previously described [68].

As positive controls, the following peptides were used: GYPKDGNAFNNLDRI (*Clostridium tetani*; G20), KEVPALTAVETGATN (Poliovirus; G21), YPYDVPDYAGYP YDV (Hemagglutinin, Influenza; G22), GDFIDYEELREQLGG (Influenza A virus H3N2; G23), and YPGEFADYEELREQL (Influenza A virus Jakarta H1N1; G24).

### 4.3. Screening and Measurement of Spot Signal Intensities

Cellulose membranes (amino-PEG500-UC540; Intavis Bioanalytical Instruments, Köln, Germany) were equilibrated with TBS (50 mM Tris-buffered saline, pH 7.0) and blocked overnight at 4 °C with TBS containing 3% casein and 0.1% Tween 20 (TBS-CT). After extensive washing with TBS-T (Tris-buffered saline, 0.1% Tween 20, pH 7.0), the membranes containing the peptide libraries were incubated for 2 h with a sera pool from twenty different patients (1:250) in TBS-CT and then washed again with TBS-T.

Following, the membranes were incubated with alkaline phosphatase (AP)-labeled goat anti-human IgM (huIgM, 1:5000 in TBS-T; KPL, Gaithersburg, MD, USA; Lot #070466) or goat anti-human IgG (huIgG, 1:5000 in TBS-CT; Thermo, Lot #JUA1121836) for 1 h, washed with TBS-T, and given a final wash in CBS (50 mM citrate-buffered saline, pH 7.0). Chemiluminescent CDP-Star^®^ Substrate (0.25 mM) with Nitro-Block-II™ Enhancer (Applied Biosystems, Waltham, MA, USA) was then added to complete the reaction.

As described previously [69], chemiluminescent signals were detected using an Odyssey FC (LI-COR Bioscience, Lincoln, NE, USA). Briefly, a digital image file was generated at a resolution of 5 MP, and the signal intensities were quantified using the TotalLab TL100 software (v. 2009, Nonlinear Dynamics, Newcastle, Tyne, UK). The signal intensity (SI) used as a background was set by negative controls spotted on each membrane. Finally, a comparative analysis of the reactivity index of the spots, normalized on a dimensional hierarchical level, was conducted using the approach previously described [70].

### 4.4. Preparation of the Chimeric Peptides

Eight multi-antigen peptides (MAP4) [PP225, PP226, PP227, PP228, PP229, PP231, PP232, PP233 (15 residues)] were synthesized using the F-moc protocol and the tetrameric TentaGel-S-NH2 resin (Intavis Bioanalytical Instruments AG, Köln, Germany) (Table S1) [71]. The synthesis of the two chimeric peptides, PP230-IgG (60 residues) and PP234 (50 residues), was conducted using Tenta gel L resin (Intavis Bioanalytical Instruments AG, Köln, Germany). Constructs were synthesized on a MultiPep-1 automated peptide synthesizer (CEM Corp, Charlotte, NC, USA), employing tetrafunctional Fmoc-amino acids with TFA-labile side chain protection where necessary. Residues of the monovalent (tail’) segment, starting with bis-Fmoc Lys, were coupled using single-step protocols. After sequence assembly, F-moc groups were removed, and the peptide-resin was cleaved and deprotected with TFA/H_2_O/EDT/TIS (94:2.5:2.5:1.0 *v*/*v*) for 90 min. The peptides were precipitated with cold diethyl ether, centrifuged three times for 10 min at 4 °C, then dissolved in 10% aqueous Acetic Acid, dried, and stored as a lyophilized powder. MAP4 was dissolved in water, centrifuged at 10,000× *g* for 60 min at 15 °C, and the supernatant filtered with a Centricon 10 filter. The single peptides were utilized without prior purification, and their identity was verified by MS (MALDI-TOF).

### 4.5. Enzyme-Linked Immunosorbent Assay (ELISA)

The in-house ELISA was performed as previously described [72]. Briefly, 96-well plates (Immulon 2HB; Thermo Fisher, Waltham, MA, USA) were incubated (12 h at 4 °C) with 50 µL synthetic peptide solution (100 µg/mL in Na_2_CO_3_-NaHCO_3_ buffer, 0.1 M, pH 9.6). After three washes with PBS pH 7.2, the free sites on the plate were blocked for 90 min with 200 μL of a 2% skim milk solution in PBS containing 0.1% Tween 20, pH 7.2. After three further washes in PBS, 50 µL of patient serum (1:50) was added to the wells and the plate incubated for 90 min at 37 °C. After further washes with PBS, 50 µL of goat anti-human IgG (H + L) conjugated to alkaline phosphatase (AP) (1: 5000) (Thermo Fisher, Waltham, MA, USA) was added to the wells and the plate incubated for 90 min at 37 °C. After a series of three washes in PBS pH 7.2, the pNPP substrate was added, and the plates were incubated for 15 min. Once more, 50 µL of 3N NaOH were added, and the reading was done after 30–120 min at 405 nm on a FlexStation3 (Molecular Devices, Sunnyvale, CA, USA).

For comparison, a commercial ELISA (classic kit, Serion, Curitiba, Brazil) was used. Briefly, precoated micro test plates were incubated with patients’ sera, and after removal of unbound material, anti-human Ig-AP conjugate was allowed to react with the immune complex. After washing away excess conjugation, pNPP was added, and specific antibody binding was measured photometrically.

### 4.6. Bioinformatics and In Silico Analysis Model

The complete sequence of Omp-2a (Q44620) of *B. abortus* was retrieved from the National Center for Biotechnology Information, USA (http://www.ncbi.nlm.nih.gov, accessed on 23 January 2024). Epitope locations within the three-dimensional molecular structure of Omp-2ae were identified by generating in silico protein models using the https://aideepmed.com/I-TASSER/, accessed 10 April 2024). Models were selected based on their C-score and TM-score, and the 3D models were validated using the AlphaFold v3 database [73]. Multiple sequence alignments were performed using the programs ClustalW (http://www.ebi.ac.uk/clustalw) (accessed on 5 May 2024) and BioEdit (http://www.mbio.ncsu.edu/BioEdit/bioedit.html, accessed on 5 May 2024).

### 4.7. Statistical Analysis

ELISA tests were statistically analyzed using Med Calc software version 20.218 [74]. A statistical difference was considered significant if the *p*-value was ≤0.05. Initially, the outcomes for each peptide were reported as a reactivity index (RI), determined by the optical density (OD) ratio of a particular sample to the cut-off OD values for each test. All RI values were classified as positive (>1.00) or negative (<0.005); those with a value of 0.005 were statistically significant.

## 5. Conclusions

This study systematically identified three IgM and seven IgG epitopes within the Brucella Omp-2a protein and confirmed their immunoreactivity using sera from infected patients. Several of these epitopes exhibited strong diagnostic potential, and chimeric multi-epitope peptides demonstrated robust performance in ELISAs. The incorporation of these specific peptides/epitopes into optimized serological platforms could substantially improve the sensitivity, specificity, and overall consistency of brucellosis diagnostics, ultimately enhancing both patient management and epidemiological monitoring.

## Figures and Tables

**Table 1:** List of the immunodominant IgM and IgG epitopes identified in Omp-2a of *B. abortus* through SPOT synthesis and their predicted secondary structure (C, coil; H, helix; S, strand) based on I-TASSER * prediction (https://zhanglab.ccmb.med.umich.edu/I-TASSER; accessed on 28 December 2023).

Code	Sequence	Peptide Start	Peptide End	2nd Structure	Ig Type	Specificity
Omp-2a/1M	NNSRHDGQYGDFSDD	116	130	S + C	IgM	*Brucella* sp., *Rhodotorula* sp.
Omp-2a/2M	NGFSAVIALE	151	165	S + C	IgM	*Brucella* sp., *Bartonella tamiae*, *Ochrobactrum* sp.
Omp-2a/3M	FTITPEVSYTKFGGE	285	300	S + C	IgM	*Brucella* sp.
Omp-2a/4G	FNYTSNNSRHDGQYG	111	125	S + C	IgG	*Brucella* sp., *Rhodotorula* sp.
Omp-2a/5G	TFTGGNGFSAVIALE	146	160	S + C	IgG	*Brucella* sp., *Bartonella tamiae*, *Ochrobactrum* sp.
Omp-2a/6G	VAYDSVIEEWATKVRGDVNI	196	215	S + C	IgG	*Brucella* sp., *Pseudochrobactrum saccharolyticum*
Omp-2a/7G	NYGQWGGDWA	236	245	C + S	IgG	*Brucella* sp., *Falsochrobactrum ovis*, *B. tamiae*
Omp-2a/8G	VWGGAKFIAPEKATF	246	260	S	IgG	*Brucella* sp., *Falsochrobactrum ovis*, *Ochrobactrum anthropi*
Omp-2a/9G	HDDWGKTAVTANVAY	266	280	C + S	IgG	*Brucella* sp., *Falsochrobactrum ovis*
Omp-2a/10G	KFGGEWKDTVAEDNA	296	310	C	IgG	*Brucella* sp.

## Data Availability

All data required to evaluate the conclusions of this study are presented in the paper and/or Supplementary Materials. Additional data related to this study can be obtained from the corresponding author upon reasonable request.

## Supplementary Materials

The following supporting information can be downloaded at: https://www.mdpi.com/article/10.3390/ijms27125341/s1.
