# Supplementary material for: IgM and IgG Epitope Mapping of the Porin Outer Membrane Protein-2a from Brucella abortus: Potential Biomarkers for Detecting Exposure to Brucellosis
Source: Int J Mol Sci. 2026 Jun 13;27(12):5341. doi: 10.3390/ijms27125341 (PMC13299733; doi:10.3390/ijms27125341)
Supplement: Supplementary file 1 [file ijms-27-05341-s001.zip › ijms-3938999-supplementary.pdf]

Supplementary Materials

Table S1. List of the synthesized epitope/peptides. Glycine (G) residues in larger letters were added to standardize the length of all synthetic single peptides. For chimeric peptide PP234, glycine's were also used to separate the epitopes and ensure consistent peptide size.

Peptide Code	Sequence
#231 (Omp-2a/1M)	NNSRHDGQYGDFSDD
#232 (Omp-2a/2M)	GGGNGFSAVIALEGG
#233 (Omp-2a/3M)	FTITPEVSYTKFGGE
#225 (Omp-2a/5G)	GTFTGGNGFSAVIALE
#226 (Omp-2a/6G)	VAYDSVIEEWATKVRGDVNI
#227 (Omp-2a/7G)	GGGNYGQWGGDWAGG
#228 (Omp-2a/8G)	VWGGAKFIAPEKATF
#229 (Omp-2a/9G)	HDDWGKTAVTANVAY
#230 (231+232+233)	GGGNNSRHDGQYGDFSDDGGNGFSAVIALEGGG FTITPEVSYTKFGGEGG
#234(226+227+228+229)	VAYDSVIEEWATKVRGDVNI NYGQWGGDWA VWGGAKFIAPEKATF HDDWGKTAVTANVAY

Table S2. List of synthesized peptides covering the entire sequence of Omp-2a protein (Uniprot Database) from *Brucella abortus.* This table details the synthesized peptides covering the entire Omp-2 protein sequence, including positive controls (F3, F4, F5, F11, F12, F13) and negative controls (F9, F17). The overlapping of positive peptides is highlighted in red.

Spot	Sequence	Spot	Sequence	Spot	Sequence	Spot	Sequence
A1	MRTLKSLVIVSAALL	B19	MKRFRIVAPLALMSL	D13	ARFALMFNTNSETEL	F7	KFGGEWKDTVAEDNA
A2	SLVIVSAALLPFSAT	B20	IVAPLALMSLALAAC	D14	MFNTNSETELGTLGT	F8	WKDTVAEDNAWGGIV
A3	SAALLPFSATAFAAD	B21	ALMSLALAACETTGP	D15	SETELGTLGTYTQLR	F9	AEDNAWGGIVRFQRS
A4	PFSATAFAADAIQEQ	B22	ALAACETTGPGSGNA	D16	GTLGTYTQLRFNYTS	F10	EDNAWGGIVRFQRSF
A5	AFAADAIQEQPPVPA	B23	ETTGPGSGNAPIIAH	D17	YTQLRFNYTSNNSRH	F11	
A6	AIQEQPPVPAPVEVA	B24	GSGNAPIIAHTPAGI	D18	FNYTSNNSRHDGQYG	F12	MRRIQSIARSPIAIA
A7	PPVPAPVEVAPQYSW	C1	PIIAHTPAGIEGSWV	D19	NNSRHDGQYGDFSDD	F13	SIARSPIAIALFMSL
A8	PVEVAPQYSWAGGYT	C2	TPAGIEGSWVDPNGI	D20	DGQYGDFSDDRDVAD	F14	PIAIALFMSLAVAGC
A9	PQYSWAGGYTGLYLG	C3	EGSWVDPNGIASSFN	D21	DFSDDRDVADGGVST	F15	LFMSLAVAGCASKKN
A10	AGGYTGLYLGYGWNK	C4	DPNGIASSFNGGIFE	D22	RDVADGGVSTGKIAY	F16	AVAGCASKKNLPNNA
A11	GLYLGYGWNKAKTST	C5	ASSFNGGIFETRTTD	D23	GGVSTGKIAYTFTGG	F17	ASKKNLPNNAGDLGL
A12	YGWNKAKTSTVGSIK	C6	GGIFETRTTDTNEKL	D24	GKIAYTFTGGNGFSA	F18	LPNNAGDLGLGAGAA
A13	AKTSTVGSIKPDDWK	C7	TRTTDTNEKLAEGNY	E1	TFTGGNGFSAVIALE	F19	GDLGLGAGAATPGSS
A14	VGSIKPDDWKAGAFA	C8	TNEKLAEGNYLYLSP	E2	NGFSAVIALEQGGED	F20	GAGAATPGSSQDFTV
A15	PDDWKAGAFAGWNFQ	C9	AEGNYLYLSPQLVEI	E3	VIALEQGGEDVDNDY	F21	TPGSSQDFTVNVGDR
A16	AGAFAGWNFQQDQIV	C10	LYLSPQLVEINMRSI	E4	QGGEDVDNDYTIDGY	F22	QDFTVNVGDRIFFDL
A17	GWNFQQDQIVYGVEG	C11	QLVEINMRSIVRGTT	E5	VDNDYTIDGYMPHVV	F23	NVGDRIFFDLDSSLI
A18	QDQIVYGVEGDAGYS	C12	NMRSIVRGTTSKVNC	E6	TIDGYMPHVVGGLKY	F24	IFFDLDSSLIRADAQ
A19	YGVEGDAGYSWAKKS	C13	VRGTTSKVNCALVSP	E7	MPHVVGGLKYAGGWG	G1	DSSLIRADAQQTLSK
A20	DAGYSWAKKSKDGLE	C14	SKVNCALVSPTQLNC	E8	GGLKYAGGWGSIAGV	G2	RADAQQTLSKQAQWL
A21	WAKKSKDGLEVKQGF	C15	ALVSPTQLNCTSSAG	E9	AGGWGSIAGVVAYDS	G3	QTLSKQAQWLQRYPQ
A22	KDGLEVKQGFEGSLR	C16	TQLNCTSSAGSRFSL	E10	SIAGVVAYDSVIEEW	G4	QAQWLQRYPQYSITI
A23	VKQGFEGSLRARVGY	C17	TSSAGSRFSLTRRNA	E11	VAYDSVIEEWATKVR	G5	QRYPQYSITIEGHAD
A24	EGSLRARVGYDLNPV	C18	SSAGSRFSLTRRNAG	E12	VIEEWATKVRGDVNI	G6	YSITIEGHADERGTR
B1	ARVGYDLNPVMPYLT	C19		E13	ATKVRGDVNITDRFS	G7	EGHADERGTREYNLA
B2	DLNPVMPYLTAGIAG	C20	MNIKSLLLGSAAALV	E14	GDVNITDRFSVWLQG	G8	ERGTREYNLALGQRR
B3	MPYLTAGIAGSQIKL	C21	LLLGSAAALVAASGA	E15	TDRFSVWLQGAYSSA	G9	EYNLALGQRRAAATR
B4	AGIAGSQIKLNNGLD	C22	AAALVAASGAQAADA	E16	VWLQGAYSSAATPNQ	G10	LGQRRAAATRDFLAS
B5	SQIKLNNGLDDESKF	C23	AASGAQAADAIVAPE	E17	AYSSAATPNQNYGQW	G11	AAATRDFLASRGVPT
B6	NNGLDDESKFRVGWT	C24	QAADAIVAPEPEAVE	E18	ATPNQNYGQWGGDWA	G12	DFLASRGVPTNRMRT
B7	DESKFRVGWTAGAGL	D1	IVAPEPEAVEYVRVC	E19	NYGQWGGDWAVWGGA	G13	RGVPTNRMRTISYGN
B8	RVGWTAGAGLEAKLT	D2	PEAVEYVRVCDAYGA	E20	GGDWAVWGGAKFIAP	G14	NRMRTISYGNERPVA

B9	AGAGLEAKLTDNILG	D3	YVRVCDAYGAGYFYI	E21	VWGGAKFIAPEKATF	G15	ISYGNERPVAVCDAD
B10	EAKLTDNILGRVEYR	D4	DAYGAGYFYIPGTET	E22	KFIAPEKATFNLQAA	G16	ERPVAVCDADTCWSQ
B11	DNILGRVEYRYTQYG	D5	GYFYIPGTETCLRVH	E23	EKATFNLQAAHDDWG	G17	VCDADTCWSQNRRAV
B12	RVEYRYTQYGNKNYD	D6	PGTETCLRVHGYVRY	E24	NLQAAHDDWGKTAVT	G18	TCWSQNRRAVTVLNG
B13	YTQYGNKNYDLAGTT	D7	CLRVHGYVRYDVKGG	F1	HDDWGKTAVTANVAY	G19	SQNRRAVTVLNGAGR
B14	NKNYDLAGTTVRNKL	D8	GYVRYDVKGGDDVYS	F2	KTAVTANVAYQLVPG	G20	GYPKDGNAFNNLDRI
B15	LAGTTVRNKLDTQDI	D9	DVKGGDDVYSGTDRN	F3	ANVAYQLVPGFTITP	G21	KEVPALTAVETGATN
B16	VRNKLDTQDIRVGIG	D10	DDVYSGTDRNGWDKG	F4	QLVPGFTITPEVSYT	G22	YPYDVPDYAGYPYDV
B17	KLDTQDIRVGIGYKF	D11	GTDRNGWDKGARFAL	F5	FTITPEVSYTKFGGE	G23	GDFIDYEELREQLGG
B18		D12	GWDKGARFALMFNTN	F6	EVSYTKFGGEWKDTV	G24	YPGEFADYEELREQL

Table S3. Values of the statistical analysis of ELISA and ROC data. The area under the curve (AUC) serves as a measure of the test's overall accuracy.

Peptides	AUC	Std. Error	95% CI	P value
PP225	0,9649	0,02203	0,9217 to 1,000	<0,0001
PP226	0,9853	0,01156	0,9626 to 1,000	<0,0001
PP227	0,9928	0,005387	0,9822 to 1,000	<0,0001
PP228	0,9892	0,007279	0,9749 to 1,000	<0,0001
PP229	0,9047	0,0359	0,8343 to 0,9751	<0,0001
PP230	0,968	0,02044	0,9279 to 1,000	<0,0001
PP231	0,7944	0,04821	0,6999 to 0,8889	<0,0001
PP232	0,6131	0,06312	0,4894 to 0,7368	0,0676
PP233	0,6284	0,06079	0,5092 to 0,7475	0,0381
PP234	0,7689	0,04954	0,6718 to 0,8660	<0,0001
Kit Serion ELISA IgG	0,9452	0,02119	0,9037 to 0,9867	<0,0001

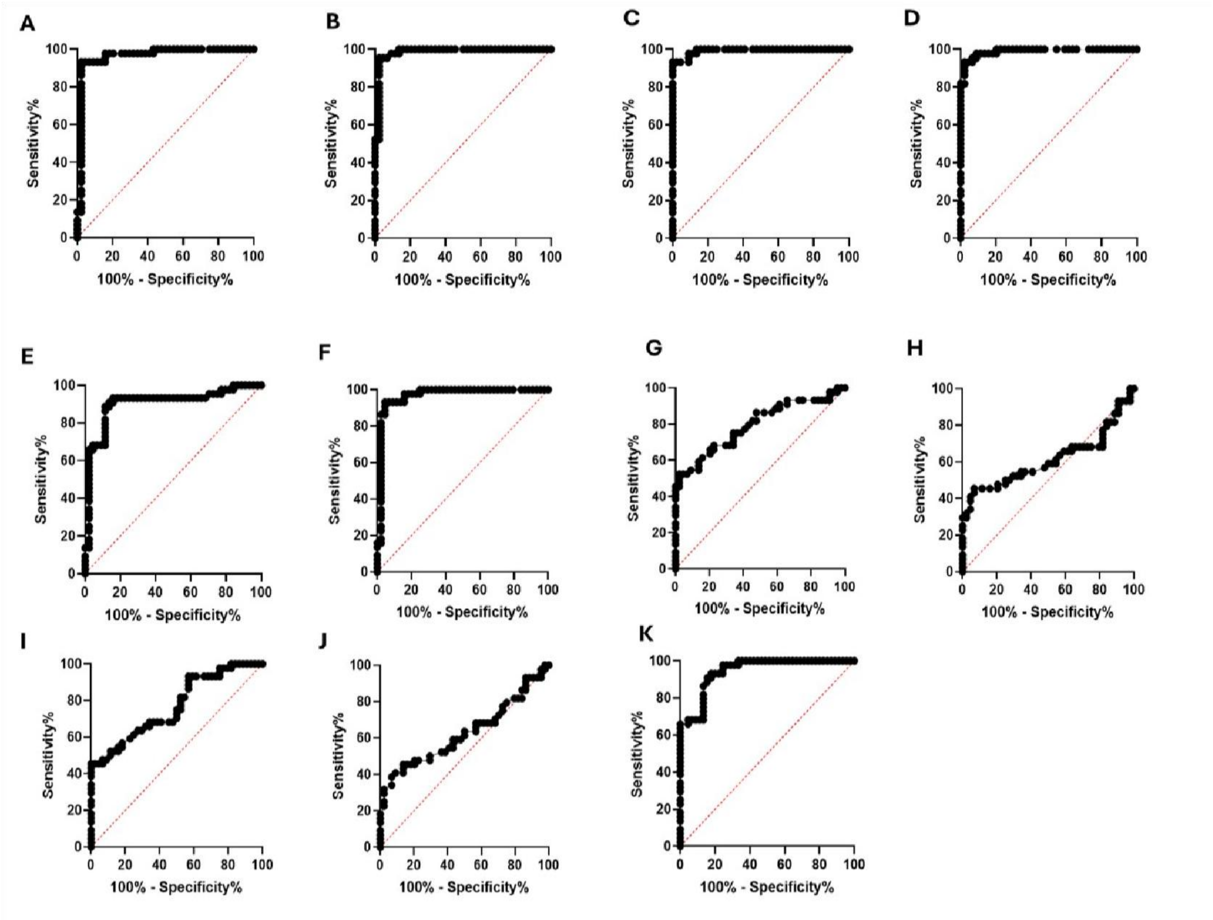

Figure S1. ROC curves illustrate the sensitivity and specificity of the evaluated peptides. Panels **A** to **J** correspond to peptides PP225 (**A**), PP226 (**B**), PP227 (**C**), PP228 (**D**), PP229 (**E**), PP230 (**F**), PP231 (**G**), PP232 (**H**), PP233 (**I**), and PP234 (**J**), respectively. Panel **K** represents the commercial Serion ELISA classic IgG kit used as a reference.
